# Checkpoint inhibitors in melanoma and early phase development in solid tumors: what’s the future?

**DOI:** 10.1186/s12967-017-1278-5

**Published:** 2017-08-08

**Authors:** Paolo A. Ascierto, Grant A. McArthur

**Affiliations:** 1 Istituto Nazionale Tumori IRCCS Fondazione “G. Pascale”, Via Mariano Semmola, 80131 Naples, Italy; 2 Peter MacCallum Cancer Centre, Melbourne, VIC Australia; 3 University of Melbourne, Parkville, VIC Australia

**Keywords:** Anti-PD-1, Anti-PD-L1, Combination therapy, Immunotherapy, Melanoma

## Abstract

Anti-programmed death (PD)-1 and PD-ligand (L)-1 checkpoint inhibitors have revolutionized the therapy of several cancers. Immunotherapy of cancer can offer long-term durable benefit to patients, is active regardless of tumour histology, has a unique immune-related safety profile, and can be used in combination with other cancer treatments. In addition, recent research has shown that immune-based therapy can be used as adjuvant therapy, that outcomes may be influenced by dose, and that clinical activity is observed in patients with brain metastases. Despite our increased understanding of these agents, there are still several important questions that need to be answered. These include strategies to overcome primary and acquired resistance, the influence of mutational status on treatment outcomes, the optimal duration of treatment, and the need to identify novel combination regimens that offer increased anti-tumour potency and/or reduced toxicity. Here we review recent developments in these areas, with particular focus on new data reported at the 2017 ASCO Annual Meeting.

## Background

Anti-programmed death (PD)-1 and PD-ligand (L)-1 checkpoint inhibitors have revolutionized the therapy of several cancers, initially in advanced melanoma but now in several other tumour types, including non-small-cell lung cancer (NSCLC), squamous cell carcinoma of the head and neck (SCCHN), urothelial bladder cancer, renal cell cancer and Hodgkin’s lymphoma.

In the past few years, we have learned that immuno-oncology (I-O) therapy can offer long-term benefit to patients, is active regardless of tumour histology, has a unique safety profile, and can be used in combination with other cancer treatments (e.g. chemotherapy, targeted therapy, radiation). Most recently, research has shown that I-O therapy can be used as adjuvant therapy [1], outcomes may be influenced by dose [2], and that clinical activity is observed in patients with brain metastases [3].

However, there are still several important questions that need to be answered. These include strategies to overcome primary and acquired resistance, the influence of mutational status on treatment outcomes, the optimal duration of treatment, and the need to identify novel combination regimens that offer increased anti-tumour potency and/or reduced toxicity. Here we review recent developments in these areas, with particular focus on new data reported at the 2017 ASCO Annual Meeting.

## Treatment resistance

Although anti-PD-1/PD-L-1 therapy has improved clinical outcomes, the majority of patients still fail to respond, due to intrinsic resistance as determined by standard oncology response criteria [4]. Also, among those patients who do respond, disease progression can occur due to acquired resistance [5]. Very little data from humans actually exists and some data comes from pre-clinical models. However, previous reports in humans have indicated that the most important mechanisms of primary resistance might include the loss of major histocompatibility complex (MHC), an increase of the number of regulatory cells into the tumour microenvironment (e.g. regulatory T cells [Tregs], myeloid derived suppressor cells [MDSCs], tumour-associated macrophages [TAMs], etc.), an increase of the production of immunosuppressive cytokines (such as interleukin [IL]-10 and TGF-β), and the upregulation of checkpoint molecules (such as LAG3). Recently, biopsy samples from four patients with metastatic melanoma who had had an initial response to anti-PD-1 therapy with pembrolizumab followed by disease progression suggested that mutations in genes encoding for interferon receptor-associated Janus kinase (JAK) 1, JAK2 or β2-microglobulin (a necessary constituent of the MHC class I complex) may be involved in the development of acquired resistance [6]. Similar mechanisms have also been described for resistance to anti-CTLA-4 [7].

## Mutational status

Immuno-oncology can be effective regardless of tumour histology and mutational status. Data from the Italian ipilimumab expanded access programme clearly showed no difference in term of overall survival (OS) between patients who harbored the BRAF mutation and those who were BRAF wild-type [8]. However, results from two recent clinical trials, the CA184-169 study of ipilimumab 10 mg/kg versus ipilimumab 3 mg/kg [2] and the CheckMate 067 trial of ipilimumab plus nivolumab versus ipilimumab or nivolumab monotherapy [9], showed better outcomes with, respectively, ipilimumab 10 mg/kg and the combined ipilimumab plus nivolumab regimen in BRAF-mutated patients. Whilst it is possible that could simply be attributed to the limitations of subgroup analyses with imbalance between the groups, we speculate that new biological data in NSCLC coupled with recently published data from melanoma provide a plausible explanation.

Second-line treatment with anti-PD-1/PD-L1 therapy is ineffective in patients with EGFR mutations in NSCLC [10–12]. The EGFR mutation is responsible for low interferon (IFN)-γ signature and higher expression of CD73, which metabolisises the conversion of AMP to adenosine [13]. Adenosine is highly immunosuppressive with several effects on immune cells and the tumor microenvironment. Streicher et al. reported that median CD73 expression was increased 10 fold compared to wild-type cell lines in EGFR-mutant NSCLC cell lines, while anti-EGFR tyrosine kinase inhibitor treatment resulted in dose-dependent inhibition of CD73 expression, suggesting a causal relationship between the EGFR pathway and CD73 expression [14]. In addition, EGFR-mutant tumours had ≥2 fold increased expression of CD73 compared to wild-type in NSCLC adenocarcinoma patients. These EGFR mutants had significantly lower levels of a IFN-γ signature, previously reported to be associated with enhanced benefit from the anti-PD-L1 agent, durvalumab [13]. High CD73 expression is also associated with low PD-L1 expression [15].

**Fig. 1:**
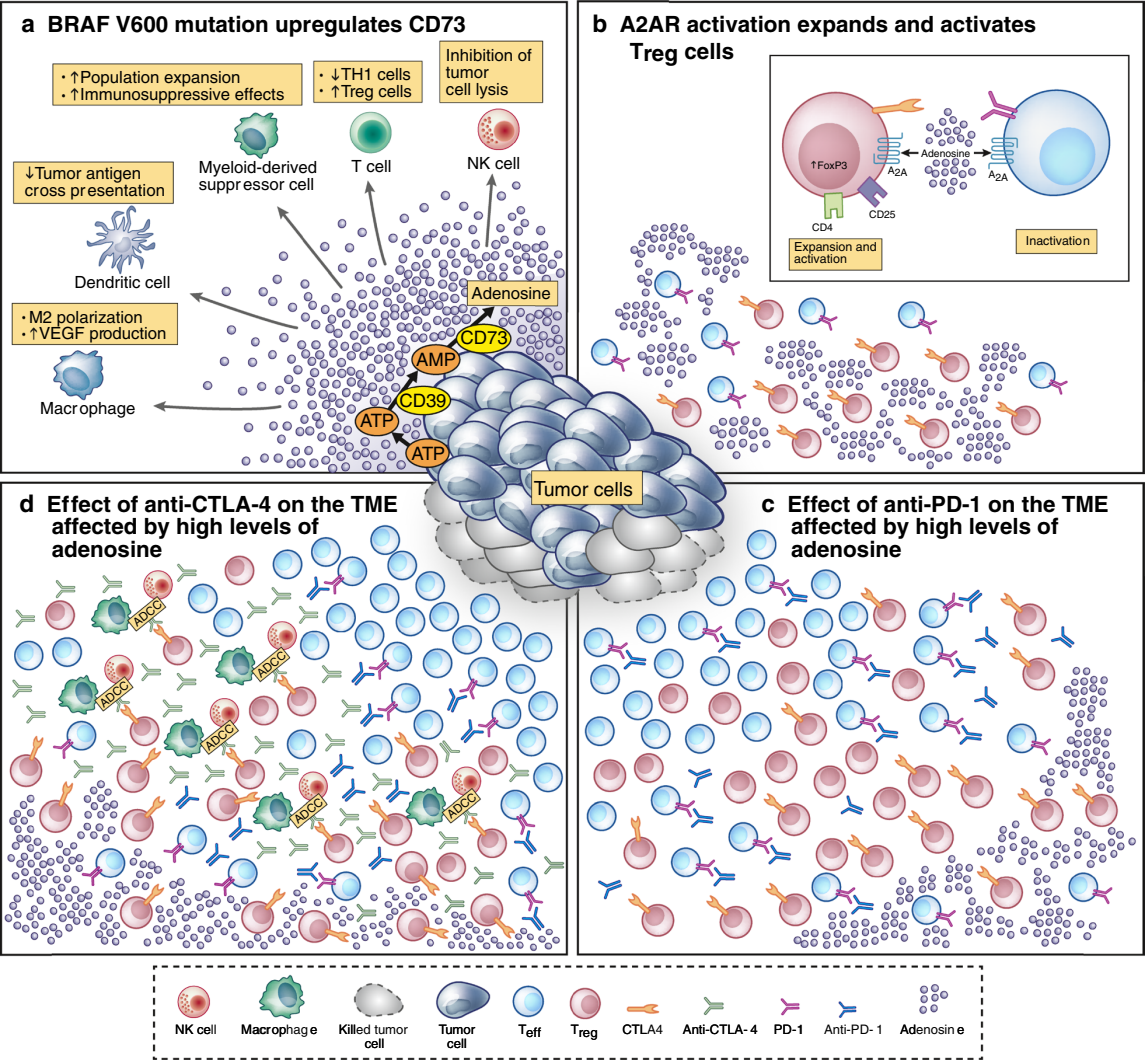
Hypothetical model about how BRAFV600 mutation in melanoma cells could affect the tumor microenvironment and response to ipilimumab and combination of ipilimumab and nivolumab. **a** The BRAFV600 mutation is able to upregulate the expression of CD73 on the melanoma cells [16] which is responsible of the increase of adenosine into the tumor microenvironment (TME). Adenosine is strongly immunosuppressive affecting almost all the immune cells. In non small cell lung cancer (NSCLC) the EGFR mutation is able to make a similar condition [14]. **b** The detailed action of adenosine on T regulatory cells (Treg), and T effector cells (Teff). Adenosine, binding the A_2A_ receptor (A2AR), is able to expand and activate Treg cells increasing the nuclear expression of FoxP3; at the same time inactivating Teff cells through the increase of CTLA-4 and PD-1 expression, and decrease of IL-2 production and CD25 expression, proliferation, T_H_1 and T_H_2 development, T_H_17 generation. The result of these pleotropic effects of adenosine is a “stuck” TME with high number of activated Treg cells and exhausted T cells. **c** In the immune suppressed TME induced by adenosine, anti-PD-1 is able to remove the blockade caused by the activation of PD-1/PD-L1 pathway and Teff cells can kill melanoma cells. In NSCLC, the EGFR mutation is also responsible for the low expression IFN-γ signature [14] contributing at the low effect of anti-PD-1 in this group of patients. **d** Higher dosage of ipilimumab (low dosage of ipilimumab does not seem to affect the TME [8]), or the addition of ipilimumab to nivolumab, may trigger an ADCC mechanism of action removing the activated Treg cells and improving the action of ipilimumab as single agent or in combination with nivolumab where there is the additional important activation of Teff cells mediated by anti-PD-1

A recently published study from Young and colleagues indicates BRAF mutation may also create CD73-dependent immune suppression in melanoma [16]. These investigators found a possible association between higher expression of CD73 and BRAF mutation. They were also able to demonstrate enhanced efficacy in preclinical in vivo models when adenosine signalling was inhibited in addition to combined BRAF and MEK inhibition. Collectively these results suggest that over-expression of CD73 in EGFR-mutant NSCLC patients and BRAF-mutant melanoma may, in part, explain the reduced benefits from anti-PD-1/PD-L1 therapy in EGFR-mutant NSCLC and the benefits from higher doses of ipilimumab or the combination of ipilimumab and nivolumab in BRAF-mutant melanoma (Fig. 1). These data set the scene for evaluating inhibition of CD73 with anti-PD-1/PD-L1 therapy in patients with oncogene driven cancers.

## Duration of treatment

Currently, the optimal duration of therapy with anti-PD-1/PD-L1 agents remains unknown. In melanoma the standard of care has switched from ipilimumab (with which only four cycles of treatment can provide a long-term benefit of 10 years for 20% of patients) [17] to anti PD-1/PD-L1 therapy, which is recommended until disease progression or occurrence of toxicity. However, it is not know if a shorter duration of PD-1/PD-L1 therapy might be equally effective. In 2016, data from a cohort of 61 patients in complete remission who stopped treatment with pembrolizumab at a median of almost 2 years showed that, at a median follow-up of about 10 months from stopping pembrolizumab therapy, only two patients had disease recurrence [18].

Robert et al. reported data from the KEYNOTE-006 study that showed a durable response [19]. At a median follow-up of nearly 3 years, 33-month OS with pembrolizumab compared to ipilimumab was 50% versus 31% and PFS was 39% versus 14%. In 104 patients who stopped pembrolizumab treatment after 2 years as per the study protocol, the estimated PFS was 91% at a median follow-up of 9.7 months after completing 2 years of pembrolizumab. Importantly, it was not just patients with complete responses who maintained PFS after stopping therapy, with a PFS rate of 91% in patients with partial responses and 83% in patients with stable disease (compared with 95% of patients with complete responses). This finding may be important in helping define the duration of therapy that is needed, with the possibility of shorter treatment and the associated benefits for patients as well as reduced healthcare costs.

## Brain metastases

Melanoma brain metastases are a common clinical presentation associated with poor prognosis. Surgery and/or stereotactic radiation therapy are widely used for oligometastatic disease and whole brain radiation therapy is used for extensive or leptomeningeal disease. However, these modalities have not been shown to improve survival and cannot impact extracranial disease and are associated with significant early and late neurotoxicity.

Experience with immunotherapy for patients with melanoma brain metastases is limited but two ongoing studies were reported at ASCO 2017. In the phase II CheckMate 204 study, nivolumab plus ipilimumab had a high intracranial ORR of 55% (21% complete responses) at a median follow-up of 9.2 months in 75 patients [20]. Median duration of response was not reached. Six-month PFS was 67% with median PFS was not reached. The safety profile of the combination was consistent with that in patents without brain metastases, with no unexpected neurologic safety signals. As in patients without brain metastases the benefits of the combination of ipilimumab and nivolumab need to be balanced with the greater toxicity when being compared to monotherapy with nivolumab. Similarly, in the phase II ABC trial in 76 patients with melanoma brain metastases and no previous checkpoint inhibitor treatment, intracranial ORR was 42% with combined ipiliumumab plus nivolumab versus 20% with nivolumab alone [21]. Median PFS with the combination was 4.8 months with 6-month PFS of 46%. Median duration of response was not yet reached. In drug treatment-naïve patients, intracranial ORR was 50% with combined treatment (21% with nivolumab alone). Intracranial ORR was 16% in patients previously-treated with BRAF/MEK inhibitors. Patients with symptomatic or leptomeningeal metastases or who had previous local therapy responded poorly to nivolumab alone.

**Fig. 2:**
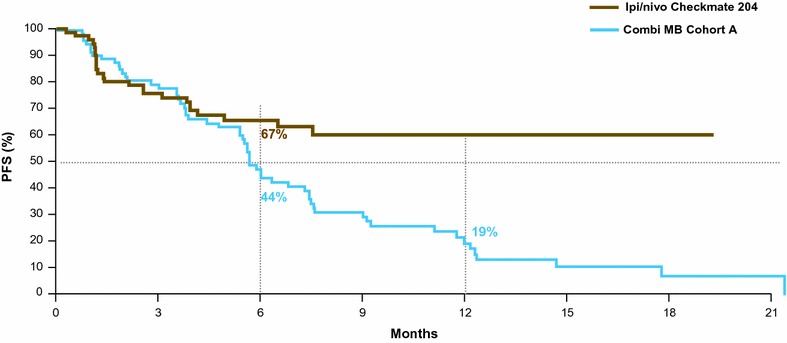
Progression free survival curves from the most recent studies on advanced melanoma patients with brain metastases. This figure shows the PFS curves from two of the most recent clinical studies in advanced melanoma patients with brain metastases (BM). The curves come from the checkmate 204 study, a phase 2 study with the combination of ipilimumab and nivolumab in advanced melanoma patients with BM [20], and from combi-MB, a phase 2 study with the combination of dabrafenib/trametinib that both reported overall PFS in patients without previous treatment for brain metastases [22]. The intent of this figure is not to directly compare results from different trials, but highlight the possibility of immunotherapy to reach, even in this group of patients with a difficult disease to treat, long-term benefit (the high and flattening tail of the curve)

Other studies in patients with melanoma brain metastases have shown clinical activity with targeted therapies. In the COMBI-MB trial, dabrafenib plus trametinib in 125 patients with BRAF V600-mutant melanoma brain metastases had a promising intracranial response rate of 58% and median duration of intracranial response of 6.5 months [22]. It is possible that targeted therapy may result in more responses but the duration of response may be lower when compared with I-O (Fig. 2).

## Novel combinations

**Fig. 3:**
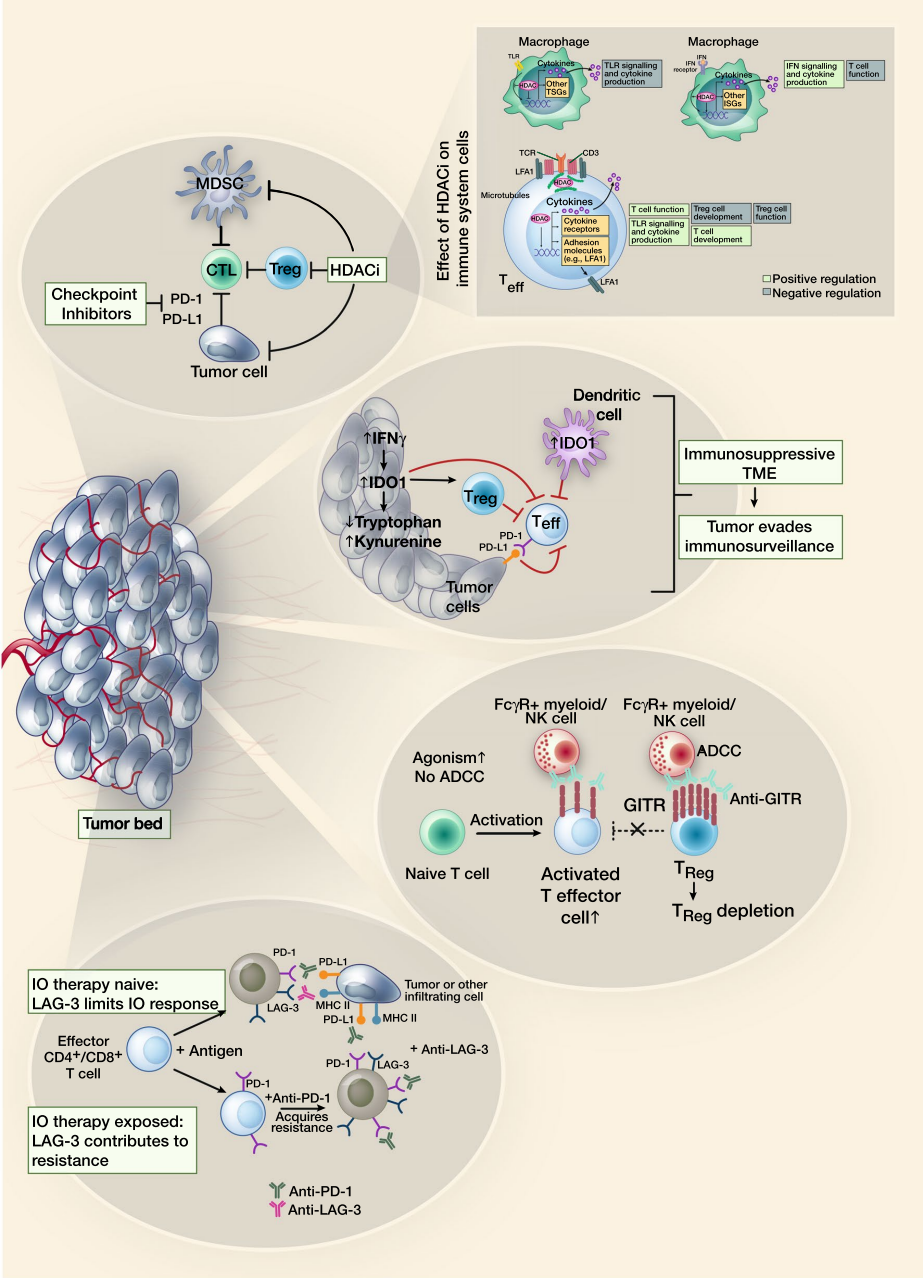
New emerging pathways for future combination with anti-PD-1/PD-L1 compounds

The potential of anti-PD-1s in combination with other novel agents is also being explored. Figure 3 summarizes the proposed mechanism of action of the recent emergent compounds. The impetus behind the development of novel combinations is to identify regimens that can overcome primary or acquired resistance to anti-PD-1/PD-L1 and/or reduce toxicity compared to combination therapy with anti-CTLA-4 and anti-PD-1. Two studies reported appeared to confirm a potential role for the indoleamine-pyrrole 2,3-dioxygenase (IDO) inhibitor epacadostat in combination with an anti-PD-1 agent.

Epacadostat was investigated in combination with pembrolizumab, in the phase I/II ECHO-202/KEYNOTE-037 trial. In 38 patients with SCCHN, ORR was 34% and DCR was 61%; ORR increased to 39% and DCR to 65% in 31 patients with only 1–2 lines of prior therapy [23]. Responses were observed regardless of PD-L1 expression or human papillomavirus (HPV) association. In the same trial, an ORR of 35% and DCR of 53% was observed In 40 patients with advanced urothelial carcinoma [24]. In this cohort, a higher response rate was observed in PD-L1-positive patients, although responses were still observed in PD-L1-negative patients. In 40 patients with NSCLC, ORR was 35% and DCR was 60% [25]. Epacadostat and pembrolizumab also showed clinical activity in patients with renal cell carcinoma (n = 33) and 0–1 prior lines of treatment, with an ORR of 47% and DCR of 58% [26]. In patients with advanced triple-negative breast cancer (n = 39) or ovarian cancer (n = 37), antitumour activity was similar to previously reported pembrolizumab monotherapy [27].

The combination was generally well tolerated across patient cohorts with a similar safety profile as pembrolizumab monotherapy, although there was a higher incidence of grade 3–4 rash [28]. Phase III studies of epacadostat plus pembrolizumab has completed accrual in melanoma and are planned in patients with SCHHN, NSCLC, urothelial carcinoma and renal cell carcinoma.

In the ECHO-204 study, patients with advanced solid tumors were treated with epacadostat (100 or 300 mg twice-daily) in combination with nivolumab and included 40 patients with treatment-naïve advanced melanoma [29]. All six patients receiving epacadostat 100 mg had a response while the ORR with the 300 mg dose was 56% (19/34). All responses were ongoing with a median duration of response of 16+ weeks (range <1+ to 41+) weeks.

Epacadostat plus nivolumab has also shown clinical activity and good tolerability in patients with other advanced solid tumours. As part of the ECHO 204 trial reported above, ORR was 23% and disease control rate (DCR) was 61% by modified Response Evaluation Criteria in Solid Tumors (mRECIST) in patients with previously-treated SCCHN. However, the combination with nivolumab did not demonstrate an efficacy signal in unselected populations of patients with refractory ovarian and colorectal cancer. Epacadostat plus nivolumab was generally well tolerated in patients with advanced solid tumours.

Another potential new treatment, entinostat, is a selective histone deacetylase (HDAC) inhibitor shown to enhance immune checkpoint inhibitor activity in preclinical studies. Preliminary data suggested that entinostat in combination with pembrolizumab showed promising activity in patients (n = 13) refractory to previous treatment with checkpoint inhibitors [30]. However, toxicity was high with 62% of patients reporting treatment-related grade 3–4 adverse events.

Similarly, addition of the anti-lymphocyte activation gene-3 (anti-LAG-3) agent BMS-986016 to nivolumab showed encouraging initial efficacy and a similar safety profile to nivolumab monotherapy in 55 patients with advanced melanoma who had disease progression on or after anti–PD-1/PD-L1 therapy [31]. Sixty-seven percent of patients had M1c disease, 38% had elevated lactate dehydrogenase (LDH) and 15% had very elevated LDH, while 76% were heavily pretreated with more than two previous therapies and 40% had progressive disease as best response to previous anti-PD-1 treatment. In this challenging cohort, ORR was 13%, with a 20% response rate in patients with LAG-3 expression ≥1% versus only 7% in LAG-3-negative (<1%) patients. Expression of PD-L1 had no impact on response. Among six partial responses, three were in resistant patients and two in refractory patients. The safety profile was comparable to that of nivolumab monotherapy. These results are of particular interest in that, if the role of LAG-3 as a predictive biomarker is confirmed by future studies, this type of combination may be advantages over ipilimumab plus nivolumab or other combination regimens, allowing the personalization of immune-oncology based on the expression of different biomarkers.

Nivolumab has also been assessed in combination with the glucocorticoid-induced tumor necrosis factor receptor-related gene (GITR) agonist, BMS-986156 [32]. GITR is a costimulatory activating receptor that is upregulated upon T cell activation. Antitumour activity of GITR agonists may be via increased T effector cell survival and function, reduced Treg-mediated suppression of T effector cells and Treg reduction through conversion to other immune cells. In a phase I/IIa study of in patients with advanced solid tumors, to date 29 patients have received BMS-986156 alone and 37 have received BMS-986156 plus nivolumab. No dose-limiting toxicities have been reported and the most common treatment-related adverse events have includes pyrexia, chills and fatigue, with all grade 1/2 except in four patients. The safety profile of the combination was similar to monotherapy. The combination showed antitumor activity in several patients, increasing proliferating (Ki67+) NK and CD8 cells in peripheral blood and increasing the activation and proliferation of CD8 memory cells. Patients who responded included heavily pretreated patients and those who had previously progressed on anti-PD-1 therapy.

Finally, there is also interest in changing the tumour microenvironment to enhance immune cell localization and activation to overcome resistance to anti-PD-1/PD-L1 therapies. These approaches include oncolytic viruses and small molecules such as toll-like receptor agonists [33] and STING-agonists. In a randomized phase II study involving 190 patients intratumoral injection of the oncolytic virus talimogene laherperepvec was combined with ipilimumab and compared with ipilimumab alone [34]. The ORR was 39% in patients receiving the combination therapy and 18% in patients receiving ipilimumab alone with acceptable toxicity in the combination arm. Future studies are required to define the activity of oncolytic viruses in combination with immune checkpoint inhibitors in patients progressing on anti-PD-1/PD-L1 treatment.

## Conclusions

Data reported at ASCO 2017 has provided important new information regarding several of the outstanding questions that relate to I-O. These include new insights into possible mechanisms of resistance that might lead to new treatment combinations (e.g. anti-PD-1/PD-L1 agents with CD73 inhibitors) and the first data to show an ongoing benefit of 2 years pembrolizumab therapy almost a year after stopping treatment, with possible implications for the optimal duration of treatment with anti-PD-1 agents. More data has suggested a role for I-O in patients with melanoma brain metastases. In addition, new potential treatment combinations have been identified, including anti-PD-1 therapy in combination with an IDO inhibitor or a new anti-LAG-3 checkpoint inhibitor. Future years will hopefully see these questions more fully addressed as ongoing trials continue and new studies are initiated.
